# Concordance between ^13^C:^12^C ratio technique respect to indirect calorimetry to estimate carbohydrate and Fat oxidation rates by means stoichiometric equations during exercise. A reliability and agreement study

**DOI:** 10.14814/phy2.14053

**Published:** 2019-04-25

**Authors:** Carlos González‐Haro

**Affiliations:** ^1^ Research and Development Department Non Functional Fatigue Co Barcelona Spain; ^2^ Department of Pharmacology and Physiology School of Medicine University of Zaragoza Spain

**Keywords:** ^13^C labelled, Method of reference, Non‐metabolic CO_2_, Stoichiometric equations

## Abstract

Indirect calorimetry is a tool used routinely by sport/exercise physiologist to assess the metabolic response to training and to nutritional interventions. There are different stoichiometric equations to estimate fat (Fat_OxR_) and carbohydrates (CHO_O__xR_) oxidation rates, however there are not enough information in literature about what are the most accurate equations. The purpose of this study was to determine the concordance between indirect calorimetry and a method of reference for stoichiometric equations used to estimate Fat_OxR_ and CHO_O__xR_. Concordance between indirect calorimetry and the method of reference (^13^C to ^12^C ratio (^13^C:^12^C ratio) technique) for key stoichiometric equations was assessed in well‐trained triathletes. Subjects carried out a carbohydrate depletion‐repletion protocol, labeling the glycogen stores with ^13^C, and a laboratory test to assess the ^13^C metabolic response during a wide range of aerobic intensities during exercise. All the equations showed a narrow agreement interval (Δ) (CHO_O__xR_ nPC (protein component negligible): −0.308, 0.308, CHO_O__xR_ PC (protein component): −0.268, 0.268, Fat_OxR_ nPC and PC: −0.032, 0.032 (g·min^−1^)). Fat_OxR_ showed a similar concordance (28–32%) with CHO_O__xR_ nPC ranging from 55% to 75%, and for CHO_O__xR_ PC between 51% to 71%. None of the stoichiometric equations met a perfect agreement with the method of reference. The Jeukendrup and Wallis equation showed the best concordance for CHO_O__xR_ nPC whilst the Frayn and Ferrannini (Glu) equations had the best agreement for CHO_O__xR_ PC. All Fat_OxR_ equations showed similar concordances and they are able to be used indistinctly.

## Introduction

The human being uses chemical energy, derived from fuels, to sustain its life cycle and to produce mechanical power during daily activities. In sport/exercise, high intensity aerobic actions are supported primarily by the energy provided from carbohydrates (CHO) while submaximal intensities are supported by a fuel mixture of mainly CHO and fat. The reason lies in CHO having faster metabolic availability than the other fuels – despite the metabolic power of fat being higher. In endurance activities, when skeletal muscle and liver glycogen availability decrease, fat utilization increases in an attempt to keep up with energy demand (Spriet 2014). Concomitantly, certain amino acids (i.e. alanine or glutamine) along with other carbon based compounds (i.e. lactate, or glycerol) are also metabolized in order to keep glucose homeostasis via the glyconeogenesis pathway (Ferrannini 1988). It is the general consensus that the main determinants of fuel oxidation rate (Fuel_OxR_) are intensity and duration of exercise, however other multiple factors such as type of diet, intra‐ and extra‐ cellular environment, gender, environmental conditions and training status have a direct, but a relatively minor, influence on substrate utilization at rest and during exercise (Gonzalez‐Haro 2011, 2015).

Metabolic pathways transform chemicals to mechanical energy through a complex network, following the basic laws of thermodynamics. Fuel_OxR_ have different stoichiometric equations due to their differences in chemical composition whereby each of the substrates need specific amounts of oxygen (O_2_) when oxidized which, in turn, produce specific amounts of carbon dioxide (CO_2_) and water. The respiratory quotient (RQ) considers the carbon dioxide production ($\dot{V}$CO_2_) to oxygen consumption ($\dot{V}$O_2_) ratio ($\dot{V}$CO_2_/$\dot{V}$O_2_) for the metabolic reactions at a tissue level. Subsequently, this makes it possible to estimate the respiratory exchange ratio (RER) at pulmonary level by means of indirect calorimetry (Ferrannini 1988). A mixture of substrates used at rest, and during exercise, are estimated measuring RER and by applying the stoichiometric equations. One of the first methodologies used to calculate Fat_OxR_ and CHO_OxR_ proportions (two components), in relation to energy expenditure, is the nonprotein RQ table of Zuntz (1897) (subsequently modified by Lusk (1924)) which estimates energy equivalence of oxygen and the percentage of energy provided from CHO and fats by means of specific assumptions. These assumptions being: (1) RQ ratio considers certain metabolic processes to be negligible (i.e. glyconeogenesis from proteins, ketone body formation and lipogenesis), (2) that RER reflects RQ where there are no changes of bicarbonate reserves in the body, (3) there is no differentiation of CHO source ((i.e. muscle or liver glycogen, glyconeogenesis (from trioses, amino acids or glycerol) or glucose from CHO ingested before/during measurement)) and (4) there is no differentiation in fat source or type (i.e. adipose tissue or muscle trialglycerol stores, lipoproteins or fatty acids ingested before/during measurement) (Frayn 1983; Peronnet et al. 1990). In regards to protein metabolism, there have been authors (Du Bois 1924; Michaelis 1924; Jungas et al. 1992) who have included protein metabolism on fuel_OxR_ estimation by measuring urine nitrogen excretion. The protein RQ chart (i.e. three components) estimates energy expenditure (EE) and the %$\dot{V}$O_2_ derived from CHO, fat and proteins, measuring: (1) urinary/sweat nitrogen, (2) $\dot{V}$O_2_ and (3) RER (without deducting the proportion corresponding to proteins oxidation rate (P_OxR_)). In recent decades, some authors have developed different stoichiometric equations to estimate FAT_OxR_, CHO_OxR_ and P_OxR_ average based on various fuel compositions (Jeukendrup and Wallis 2005).

Indirect calorimetry is currently a routine tool that is often used to measure EE and fuel selection for the majority of physiological laboratories around the world (52 reviews listed in the US National Library of Medicine's PubMed resource containing the text words *indirect calorimetry, exercise, review*, up to January 2019). Indirect calorimetry and stoichiometry does present some limitations when estimating fuel selection as it relies on the assumption that $\dot{V}$O_2_ and $\dot{V}$CO_2_ reflect the gas exchange at tissue level. However, while O_2_ measurements are typically reliable, mainly due to limited stores in the human body, large CO_2_ stores do exist (Frayn 1983). Taking this into account, $\dot{V}$CO_2_ excreted by breath is only reliable when the bicarbonate pool is stable – typically at rest and during low intensities of exercise. For mild to high intensities of exercise, hydrogen ion concentration [H^+^] increases while being buffered by bicarbonate [HCO_3_^−^]. This process excretes non‐metabolic CO_2_ by breath which produces a breathing $\dot{V}$CO_2_ overestimation, when measured by indirect calorimetry, and this leads to stoichiometric equations overestimating CHO_OxR_ and underestimate FAT_OxR_ (Barstow et al. 2018).

Since the proposed adjustment of Zuntz (1897), and those by Jeukendrup and Wallis (2005), researchers have tried to improve fuel_OxR_ accuracy estimation by deriving coefficients equations based on different fuel types and mixtures. One of the most accurate methodologies to estimate fuel oxidation rates is the CO_2_ labeled technique involving the administration of ^13^C‐ or ^14^C‐enriched substrates. After the ingestion of ^13^C labelled CHO the measurement of isotopes ^13^C to ^12^C ratio (^13^C:^12^C ratio), under known $\dot{V}$CO_2_ conditions, enables the calculation of the amount of ingested glucose being oxidized (Lefebvre 1985). Romijn et al. (1992) applied a mixed ^13^C:^12^C ratio technique (plasma and breath) to measure the non‐metabolic CO_2_ and subsequently estimate substrate oxidation rates independent of *inaccurate* $\dot{V}$CO_2_ measures as previously described in regards to the indirect calorimetry approach. However, indirect calorimetry, and stoichiometric equations, are still routinely used to study the physiological adaptations to exercise and there have been few attempts to investigate the agreement between the different stoichiometric equations in comparison to a reference method, and doing so would help to address the most important limitations of indirect calorimetry in estimating fuel supply during exercise (Romijn et al. 1992). Comparing the fuel_OxR_, as measured by indirect calorimetry, and applying the stoichiometric equations to the ^13^C:^12^C ratio technique is a good theoretical approach to understand the error of measurement (in g·min^−1^) when $\dot{V}$CO_2_ is measured with routinely indirect calorimeters.

Thus, the main purpose of the present study was to determine the concordance between indirect calorimetry and the ^13^C:^12^C ratio technique as a method of reference for each of the stoichiometric equations to estimate FAT_OxR_ and CHO_OxR_ during the exercise. A second purpose was to determine the influence of the protein component on stoichiometric equations during short‐term endurance‐based exercise.

## Methods

### Subjects

Sixteen well‐trained triathletes (mean ± SD age 28.6 ± 1.0 years, body weight 70.1 ± 6.7 kg, body mass index 22.3 ± 2.2 kg·m^−2^, body fat 14.1 ± 6.2%, $\dot{V}$O_2max_ 55.4 ± 5.1 mL·kg^−1^·min^−1^, experience 9.1 ± 2.8 years), one of them at Olympic level, were paid to participate in this study. All of them were healthy, normoglycemic (92 ± 12 mg·dL^−1^), free of injuries and overreaching symptoms. They were informed of the protocols and purposes of the study, provided written consent prior to participating and were advised of their right to withdraw from this research at any time. This study was conducted according to the Ethical Principles for Medical Research Involving Humans and was approved by the local research ethics committee.

### Protocol

**Figure 1:**
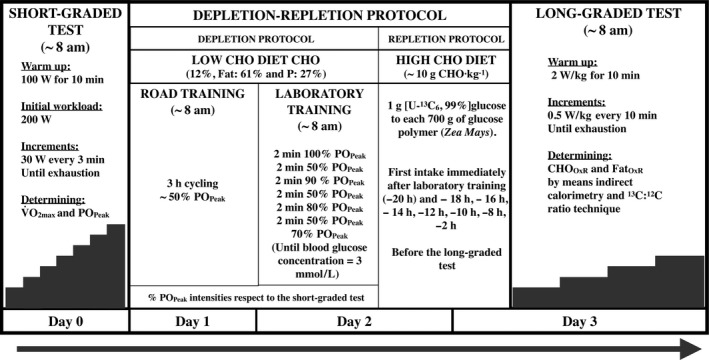
Study protocol design.

In this reliability and agreement study, all the subjects carried out a preliminary testing session (D_0_), a depletion‐repletion (^13^C enriched) CHO protocol for two consecutive days (D_1_ and D_2_), and a long‐graded laboratory test on a third consecutive day (D_3_). Each subject performed the tests in the same order and time of day (Fig. 1).

### Preliminary testing

A routine blood test was performed at ~8:00 am on D_0_. Consecutively, body mass and body fat were assessed by DXA (Lunar Prodigy Primo™, General Electric, Madison, Wisconsin, USA), data was analyzed using Lunar enCORE software. Training intensities, expressed as % peak power output (%PO_Peak_), were determined by a short graded‐laboratory test (Gonzalez‐Haro 2015). This test was carried out on a Lode Excalibur Sport cycle ergometer (Lode, Groningen, The Netherlands), which consisted of a warm‐up of 100 Watts (W) for 10 min, followed by the exercise segment starting at 200 W which was increased by 30 W every 3 min until exhaustion. Throughout the tests, cyclists freely chose their pedal rate (over 70 rpm) and rates of perceived exertion (RPE) (20‐points Borg scale) (Borg 1982) was measured at the end of the test.

### Depletion‐repletion (^13^C‐labelling) CHO protocol

Subjects followed a low CHO‐diet (CHO 12% (79 g), fat 61% (174 g) and proteins 27% (171 g), EE 2565 kcal) for two consecutive days (D_1_ and D_2_), together with 3 h road cycling training at ~50% PO_Peak_ on D_1_, and a training session under laboratory conditions on D_2_ (15 min progressive warm‐up (50–80% PO_Peak_)). Afterwards, subjects performed all repetitions possible at a given intensity (in regards to the short‐graded protocol PO_Peak_) and, once they were not able to do so, they maintained repetitions at a lower intensity: 2 min 100% PO_Peak_ + 2 min 50% PO_Peak_, 2 min 90% PO_Peak_ + 2 min 50% PO_Peak_, 2 min 80% PO_Peak_ + 2 min 50% PO_Peak_, 70% PO_Peak_, until blood glucose concentration levels dropped below 3 mmol·L^−1^ and this was measured at the end of each intensity (Accu‐Chek Compact Plus^®^, Roche Diagnostics, Basel, Switzerland). RPE was measured at the end of the protocol (Borg 1982). Similar protocols have previously provoked skeletal muscle glycogen stores to near‐complete depletion and reduced ^13^C background from the previous endogenous substrate stores (Romijn et al. 1992; Wagenmakers et al. 1993). Immediately after D_2_, subjects ingested a glucose polymer (~10 g·kg^−1^; 423 ± 3 mosmol·kgH_2_O^−1^) in equal amounts 20, 18, 16, 14, 12, 10, 8 and 2 h before the long‐graded laboratory test. The Polymer derived from 99% native maize starch (*Zea mays*) (Meritena^®^ 100, Syral Iberia SAU, Spain), with a high natural abundance of ^13^C (−11.2 *δ*‰ ^13^C vs. the reference standard Vienna Pee Dee Belemnite (VPDB), was further artificially enriched adding 1 g of [U‐^13^C_6_, 99%]glucose (Cambridge Isotope Laboratories Inc., Andover, MA, USA) (−10.8 *δ*‰) to each 700 g of glucose polymer. The taste was improved with a natural sweetener mixed by Powergym™ (Spain).

### Long‐graded laboratory tests

Subjects carried out the Gonzalez‐Haro (2015) long‐graded laboratory test on D_3_ and this test was carried out on a Lode Excalibur Sport cycle ergometer (Lode, Groningen, The Netherlands), which consisted of a warm‐up of 2.0 W·kg^−1^ for 10 min and was increased by 0.5 W·kg^−1^ every 10 min until exhaustion. Throughout the tests, cyclists freely chose their pedal rate, but above 70 rpm. RPE was measured at the end of the test (Borg 1982). Subjects drank 4 mL·kg^−1^ of water 2 h before the exercise and ~0.8 L·h^−1^ of water ad libitum*,* during the test (Gonzalez‐Haro 2015), in order to be well hydrated. Heart rate (RS800CX; Polar Electro Oy, Kempele, Finland) was monitored during the exercise. $\dot{V}$O_2_, and $\dot{V}$CO_2_ were measured and recorded (Oxycon Pro^®^, Jaeger, Germany) in real time, via the breath by breath method, throughout the test. This particular indirect calorimeter (Oxycon Pro^®^ Erich Jaeger GmbH, Hoechberg, Germany) has been previously validated against the Douglas bags method (Carter and Jeukendrup 2002; Macfarlane and Wong 2012; Foss and Hallen 2018). The Oxycon Pro^®^ was calibrated according to the instruction manual (Manual ver. 4.5, Erich Jaeger GmbH, Hoechberg, Germany) before each test. O_2_ and CO_2_ analyzers were calibrated with room air and certified calibration gases at 180 kPa (5.55% CO_2_ and 94.45% N_2_). The flow turbine (Triple V, Erich Jaeger GmbH, Hoechberg, Germany) was also calibrated with a 3‐L 5530 series calibration syringe (Hans‐Rudolph, Inc., Kansas City, USA). The calibration syringe was calibrated before testing with a motorized calibration syringe (Mod 17800, VacuMed, California, USA). The flowmeter and gas analyzers were connected to a computer that calculated the ventilatory frequency ($\dot{V}$F), tidal volume ($\dot{V}$T), fraction of O_2_ (F_E_O_2_) and CO_2_ (F_E_CO_2_) exhaled. Further, the ventilatory volume ($\dot{V}$E), respiratory exchange ratio (RER), $\dot{V}$O_2_ and $\dot{V}$CO_2_ were measured in real time, via breath by breath, throughout the test and values provided via conventional equations (Robergs 2018). Both, gas and volume, calibration were repeated until the difference between consecutive calibrations was less than 1%. The $\dot{V}$O_2_ slow component is characterized by a delayed rise in $\dot{V}$O_2_ and its magnitude was set equal to the difference in $\dot{V}$O_2_ between the fifth and the last minute at each stage, to ensure that the steady state was reached at each intensity of exercise (Robergs 2018).

Breath ^13^C:^12^C ratio and blood lactate concentrations ([La^−^]_b_) were measured at the end of basal period and each stage and at the end of the recovery period (i.e. 7th min). Gas exchange values at the end of the basal period and each stage were computed to study the concordance between indirect calorimetry and the ^13^C:^12^C ratio technique. Peak oxygen consumption ($\dot{V}$O_2Peak_), PO_Peak_, lactic threshold (LT) and individual anaerobic threshold (IAT) were calculated as published elsewhere (Gonzalez‐Haro 2015). Two 10‐mL urine samples were collected and frozen at −20°C immediately pre‐ and post‐exercise. Total urinary urea nitrogen excretion (UUNE) and the urine specific gravity (U_SG_) were determined (Kjeltec 1030 Auto Analyser, Tecator AB, Höganäs, Sweden; Urisys 1800, Roche Diagnostics, Switzerland).

The rate of glucose tissue uptake was quantified, during the long graded‐test, by infusion of [6,6‐^2^H_2_]glucose assessing the relative contribution of plasma glucose to total carbohydrate oxidation (Romijn et al. 1992). Teflon catheters (Quickcath, Baxter, Norfolk, UK) were inserted antecubital vein of one forearm for the collection of blood samples, kept patent with isotonic saline (Becton Dickinson, Drogheda, UK) containing 1 UI·L^−1^ heparin (CP Pharmaceuticals, Wrexham, UK), and into the contralateral arm for tracer infusion. Then, after a blood sample was drawn to determine background enrichment, a primed constant infusion of [6,6‐^2^H_2_]glucose (99% enriched; Isotech, Miamisburg, OH, USA) was started at the rate of 0.22 *μ*mol·kg^−l^·min^−l^ (prime 17.6 pmol·kg^−1^) and maintained during a subsequent 2‐h rest period prior to start the long‐graded test. When long‐graded test started, the rate of isotope administration was doubled to minimize changes in isotope enrichment, resulting from the stimulation of glucose production. The calibration of the infusion pumps (Asena GS, Alaris Medical Systems, Basingstoke, UK) was checked before and after use. Venous blood samples were obtained at rest and at the end of each intensity during the long‐graded test, to determine the ^13^C enrichment of plasma glucose, protein and free fatty acids. It was assumed that these enrichments represented the corresponding enrichment of glucose, protein, and fat in the remainder of the body. When [6,6‐^2^H_2_]glucose was infused, blood was taken before starting the isotope infusion. All samples were collected in 10‐mL vacutainers (Vacutainer, Becton Dickinson) containing lithium heparin at the end of the basal period and each stage. Plasma was separated by centrifugation at 4°C and frozen until further processing. To determine the plasma ^13^C:^12^C ratio of glucose, protein and fat was used the same methodology explained elsewhere (Romijn et al. 1992). The CV was <0.1%.

### Expired air collection and breath ^13^C:^12^C ratio technique

Expired air was collected by using a mouthpiece connected to a Y‐Shape™ two‐way non‐rebreathing valve, where it was attached to 6‐L non‐diffusing gas collection bags (Hans Rudolph Inc., Kansas City, Mo, USA), and was flushed twice before an expired air sample was transferred into 12‐mL evacuated glass tubes (Exetainers, Labco, High Wycombe, UK). Samples were subsequently analyzed for ^13^C:^12^C ratio by continuous‐flow isotope ratio mass spectrometry (Europa Scientific, Crewe, UK). The contents of samples and references were flushed and transported by helium carrier gas through a packed column gas chromatograph, held at 75°C. The resultant chromatographic peak then entered the isotope ratio mass spectrometry, where the isotopomers at mass‐to‐charge ratio of 44, 45, and 46 for CO_2_ were measured, and a ^13^C value was determined. The reference gas used during analysis was 3.3% CO_2_ in a helium balance with ^13^C 29.01 VPDB. The 3.3% CO_2_ mixture was prepared from a CO_2_ cylinder calibrated against NBS‐19 (^13^C value of 1.95 VPDB), an isotope reference standard distributed by the International Atomic Energy Agency, Vienna. The CV was <0.1%.

The isotopic enrichment (*δ* ^13^C) was expressed in absolute (^13^C:^12^C ratio) and relative (‰ *δ* ^13^C) values as the difference between ^13^C:^12^C ratio of the sample and a known laboratory reference standard (eq. (1)) (Craig 1957). Thereafter, ‰ *δ* ^13^C was related to the VPDB standard (‰ *δ* ^13^C VPDB) (VPDB: *δ* ^13^C = 0.0112372 = 0 *δ*‰ ^13^C)).

(1) $$\delta^{13}C_{‰\mathrm{VPDB}}=\left[\frac{{{\left(\right.}^{13}C{:}^{12}C)}_{\mathrm{sample}}-{{\left(\right.}^{13}C{:}^{12}C)}_{\mathrm{VPDB}}}{{{\left(\right.}^{13}C{:}^{12}C)}_{\mathrm{VPDB}}}\right]\cdot 10^{3}$$

Absolute ^13^C:^12^C ratio in breath (R_*b*_) is the result of the relative contributions of the ^13^C:^12^C ratios derived from combustion of carbohydrates (R_*c*_), fats (R_*f*_) and proteins (R_*p*_), which equals to 1 (*x + y + z *=* *1) (eq. (2)).

(2) $$R_{b}=xR_{C}+yR_{f}+zR_{p}$$

It is possible to calculate the relative contribution of each fuel to $\dot{V}$CO_2_ by means the following stoichiometric equations, when the source is predominantly glycogen or glucose (eq. (3)):

(3) $$\begin{array}{cc} & a)\dot{V}CO_{2\left(\mathrm{Glycogen}\right)}=0.8251\cdot \mathrm{CH}O_{\mathrm{OxR}}+1.4136\cdot \mathrm{Fa}t_{\mathrm{OxR}}+4.4176\cdot n\\ & b)\dot{V}CO_{2\left(\mathrm{Glucose}\right)}=0.7426\cdot \mathrm{CH}O_{\mathrm{OxR}}+1.4136\cdot \mathrm{Fa}t_{\mathrm{OxR}}+4.4176\cdot n \end{array}$$

where, combustion of 1 g glucose requires 0.7455 L O_2_ and produces 0.7426 L CO_2_, combustion of 1 g glycogen requires 0.8283 L O_2_ and produces 0.8251 L CO_2_ (Ferrannini 1988), 1 g fatty acid (average: C_17.2702_H_32.7142_O_2_) requires 2.0092 L O_2_ and produces 1.4136 L CO_2_ (Peronnet et al. 1990), combustion of 1 g protein (average amino acids) requires 0.9842 L O_2_ and produces 0.7931 L CO_2_ (Jeukendrup and Wallis 2005) and 1 g of UUNE is 5.57 g of protein (Jungas et al. 1992). $\dot{V}$CO_2_ (L·min^−1^), CHO_OxR_, FAT_OxR_ and n (UUNE) (g·min^−1^).

From previous assumptions, R_b_ equations were obtained for glycogen and glucose (eq. (4)). However, these equations have two unknown variables CHO_OxR_ and FAT_OxR_, which can be solved with the $\dot{V}$O_2_ equation, for glycogen and glucose:

(4) $$\begin{array}{c} a)R_{b\left(\mathrm{Glycogen}\right)}=\left[\frac{\left(0.8251\cdot {\mathrm{CHO}}_{\mathrm{OxR}}\cdot R_{c}\right)+\left(1.4136\cdot {\mathrm{Fat}}_{\mathrm{OxR}}\cdot R_{f}\right)+\left(4.4176\cdot n\cdot R_{p}\right)}{\left(0.8251\cdot {\mathrm{CHO}}_{\mathrm{OxR}}\right)+\left(1.4136\cdot {\mathrm{Fat}}_{\mathrm{OxR}}\right)+\left(4.4176\cdot n\right)}\right]\\ b)R_{b\left(\mathrm{Glucose}\right)}=\left[\frac{\left(0.7426\cdot {\mathrm{CHO}}_{\mathrm{OxR}}\cdot R_{c}\right)+\left(1.4136\cdot {\mathrm{Fat}}_{\mathrm{OxR}}\cdot R_{f}\right)+\left(4.4176\cdot n\cdot R_{p}\right)}{\left(0.7426\cdot {\mathrm{CHO}}_{\mathrm{OxR}}\right)+\left(1.4136\cdot {\mathrm{Fat}}_{\mathrm{OxR}}\right)+\left(4.4176\cdot n\right)}\right] \end{array}$$

(5) $$\begin{array}{c} a)\dot{V}O_{2\left(\mathrm{Glycogen}\right)}=0.8283\cdot {\mathrm{CHO}}_{\mathrm{OxR}}+2.0092\cdot {\text{Fat}}_{\mathrm{OxR}}+5.4820\cdot n\\ b)\dot{V}O_{2\left(\mathrm{Glucose}\right)}=0.7455\cdot {\mathrm{CHO}}_{\mathrm{OxR}}+2.0092\cdot {\text{Fat}}_{\mathrm{OxR}}+5.4820\cdot n \end{array}$$

$\dot{V}$O_2_ equations were based on the same assumptions than Eq. (3).

CHO_OxR_ and Fat_OxR_ from the ^13^C:^12^C ratio technique were derived from Equations (4) and (5), taking into account both the protein component (PC) and considering it negligible (nPC) (Equations (6) and (7)). Since these equations avoid the error introduced by the non‐metabolic CO_2_ measured with indirect calorimetry, CHO_OxR_ and Fat_OxR_ estimated from these equations were considered as the fuels of reference (i.e. method of reference) to be compared to all the stoichiometric equations listed in Table 1

(6) $$\begin{array}{cc} a)\mathrm{CH}O_{\mathrm{OxR}(\mathrm{Glycogen}\_\mathrm{PC})} & =\left[\frac{\left(0.8528\cdot \dot{V}O_{2}\cdot \left(R_{f}-R_{b}\right)\right)+\left(n\cdot \left(5.3540\cdot \left(R_{p}-R_{b}\right)\right)\right)-\left(4.6745\cdot \left(R_{f}-R_{b}\right)\right)}{\left(R_{b}-R_{c}+\left(0.7063\cdot \left(R_{f}-R_{b}\right)\right)\right)}\right]\\ b)\mathrm{CH}O_{\mathrm{OxR}(\mathrm{Glycogen}\_\mathrm{nPC})} & =\left[\frac{0.8528\cdot \dot{V}O_{2}\cdot \left(R_{f}-R_{b}\right)}{R_{b}-R_{c}+\left(0.7063\cdot \left(R_{f}-R_{b}\right)\right)}\right]\\ c)\mathrm{CH}O_{\mathrm{OxR}(\mathrm{Glucose}\_\mathrm{PC})} & =\left[\frac{\left(0.9475\cdot \dot{V}O_{2}\cdot \left(R_{f}-R_{b}\right)\right)+\left(n\cdot \left(5.9488\cdot \left(R_{p}-R_{b}\right)\right)\right)-\left(5.1938\cdot \left(R_{f}-R_{b}\right)\right)}{\left(R_{b}-R_{c}+\left(0.7063\cdot \left(R_{f}-R_{b}\right)\right)\right)}\right]\\ d)\mathrm{CH}O_{\mathrm{OxR}(\mathrm{Glucose}\_\mathrm{nPC})} & =\left[\frac{0.9475\cdot \dot{V}O_{2}\cdot \left(R_{f}-R_{b}\right)}{R_{b}-R_{c}+\left(0.7063\cdot \left(R_{f}-R_{b}\right)\right)}\right] \end{array}$$

(7) $$\begin{array}{cc} a)\mathrm{Fa}t_{\mathrm{OxR}\left(\mathrm{Glycogen}\_\mathrm{PC}\right)} & =\left(0.4977\cdot \dot{V}O_{2}-0.4123\cdot \mathrm{CH}O_{\mathrm{OxR}}-2.7285\cdot n\right)\\ b)\mathrm{Fa}t_{\mathrm{OxR}\left(\mathrm{Glycogen}\_\mathrm{nPC}\right)} & =\left(0.4977\cdot \dot{V}O_{2}-0.4123\cdot \mathrm{CH}O_{\mathrm{OxR}}\right)\\ c)\mathrm{Fa}t_{\mathrm{OxR}\left(\mathrm{Glucose}\_\mathrm{PC}\right)} & =\left(0.4977\cdot \dot{V}O_{2}-0.3710\cdot \mathrm{CH}O_{\mathrm{OxR}}-2.7285\cdot n\right)\\ d)\mathrm{Fa}t_{\mathrm{OxR}\left(\mathrm{Glucose}\_\mathrm{nPC}\right)} & =\left(0.4977\cdot \dot{V}O_{2}-0.3710\cdot \mathrm{CH}O_{\mathrm{OxR}}\right) \end{array}$$

where, $\dot{V}$O_2_: Oxygen consumption (L·min^−1^), *Rb*: Absolute ^13^C:^12^C ratio in breath, *Rc*: Relative ^13^C:^12^C ratio in breath of carbohydrates, *Rf*: Relative ^13^C:^12^C ratio in breath of fats, *Rp*: Relative ^13^C:^12^C ratio in breath of proteins, CHO_OxR_: Carbohydrates oxidation rate (g·min^−1^), *n*: UUNE (in g·min^−1^), PC: Including the protein component, and nPC: Excluding the protein component (negligible).

**Table 1:** Stoichiometric equations

For carbohydrate oxidation (g·min^−1^)			
SE_CHO__#1	Zuntz (1897), Lusk (1924)	Derived from table.	nPC
SE_CHO__#2	Du Bois (1924)	Derived from chart*.	PC
SE_CHO__#3	Brouwer (1957)	4.170·$\dot{V}$CO_2_−2.965·$\dot{V}$O_2_−0.390·p	PC, nPC
SE_CHO__#4	Frayn (1983), Ferrannini (1988) (Glu)	4.55·$\dot{V}$CO_2_−3.21·$\dot{V}$O_2_−2.87·n	PC, nPC
SE_CHO__#5	Ferrannini (1988) (Gly)	4.09·$\dot{V}$CO_2_−2.88·$\dot{V}$O_2_−2.59·n	PC, nPC
SE_CHO__#6	Peronnet and Massicotte (1991)	4.585·$\dot{V}$CO_2_−3.226·$\dot{V}$O_2_	nPC
SE_CHO__#7	Jeukendrup and Wallis (2005) (Low intensity)	4.344·$\dot{V}$CO_2_−3.061·$\dot{V}$O_2_−2.37·n	PC, nPC
SE_CHO__#8	Jeukendrup and Wallis (2005) (High intensity)	4.210·$\dot{V}$CO_2_−2.962·$\dot{V}$O_2_−2.37·n	PC, nPC
For fat oxidation (g·min^−1^)			
SE_Fat__#1	Zuntz (1897), Lusk (1924)	Derived from table.	nPC
SE_Fat__#2	Du Bois (1924)	Derived from chart.*	PC
SE_Fat__#3	Brouwer (1957)	1.718·$\dot{V}$O_2_−1.718·$\dot{V}$CO_2_−0.315·p	PC, nPC
SE_Fat__#4	Frayn (1983), Ferrannini (1988)	1.67·$\dot{V}$O_2_−1.67·$\dot{V}$CO_2_−1.92·n	PC, nPC
SE_Fat__#5	Peronnet and Massicotte (1991)	1.695·$\dot{V}$O_2_−1.701·$\dot{V}$CO_2_	nPC
SE_Fat__#6	Jeukendrup and Wallis (2005)	1.695·$\dot{V}$O_2_−1.701·$\dot{V}$CO_2_−1.77·n	PC, nPC

SE_CHO_, Stoichiometric equation for CHO; SE_Fat_, Stoichiometric equation for fat; n, UUNE; p, protein oxidation; Gly, glycogen; Glu, glucose; Low intensity, 40–50%$\dot{V}$O_2max_; High intensity, 50–75%$\dot{V}$O_2max_; PC, including the protein component; nPC, without the protein component (negligible). *percentage of calories coming from proteins where estimated (Lusk 1924) and rate of CHO_OxR_ and Fat_OxR_ were calculated from EE (kcal) following the coefficients of 1 g glucose = 3.74 kcal, 1 g glycogen = 4.15 kcal (Ferrannini 1988), 1 g fat (fatty acid average: C_17.2702_H_32.7142_O_2_) = 9.75 kcal (Peronnet and Massicotte 1991).

### Data and statistical analyses

Statistical analyses and plots were performed with the free open‐source software R (R Development Core Team 2018) running on Linux Mint 19 Cinammon. All data was presented as mean ± SE and 95% CI. Normality was determined with the Shapiro‐Wilk test. Minimum sample size, for agreement studies (Liao 2010), required for the results to have the appropriate precision was 31 measurements, for a discordance rate *α = *0.05 and a tolerance probability *ß *=* *80%. Liao and Capen (2011) concordance plots for each pair of equations (indirect calorimetry vs. ^13^C:^12^C ratio technique) were assessed by means of the R code *Liao‐Capen modified Bland‐Altman approach* (Bassani 2012), according to the following error model (Eqn. (4)):

(8) $$Y_{i}=Y_{i}^{0}=a_{0}+b_{0}\cdot X_{i}^{0}+\varepsilon_{i};X_{i}=X_{i}^{0}+\delta_{i}$$

Hereafter, the Deming regression was determined by calculating measurement error model components (*a*_0_, *b*_0_, *S*_*xx*_, *S*_*yy*_, *S*_*xy*_, and *σ* ^2^) in order to determine the agreement intervals (Δ) according to the bias (fixed or proportional) (Liao and Capen 2011). Confidence intervals, for the proportions, were computed using the Clopper‐Pearson exact confidence interval method. To assess the agreement, a threshold k of 5% was chosen as the maximum number of pairs that it is accepted to lie outside the estimated agreement interval. By choosing a value of *k* = 0.05*154 ≈ 8 pairs, the perfect agreement is ~95%. Thereafter, polynomial regressions to adjust the CHO_OxR_ and Fat_OxR_ for each stoichiometric equation to the method of reference were performed. Taking into account the best fit equation (*R* ^2^), the Deming regression was again determined as well as the Liao and Capen (2011) agreement, as explained above, between the adjustment model respect to the method of reference. Comparisons for non‐parametric data were performed via Friedman and Wilcoxon tests. Statistical significance was set at *P *<* *0.05.

## Results

For both the short‐graded and long‐graded laboratory test, RPE was high (19.2 ± 0.5 and 19.6 ± 0.6, respectively), and although there were no significant differences found this was even higher at the end of the depletion protocol (19.9 ± 0.2). All subjects reached the criterion set using the depletion CHO protocol (blood glucose < 3 mmol·L^−1^).

### ^13^C‐enrichment (*δ* ^13^C)

**Figure 2:**
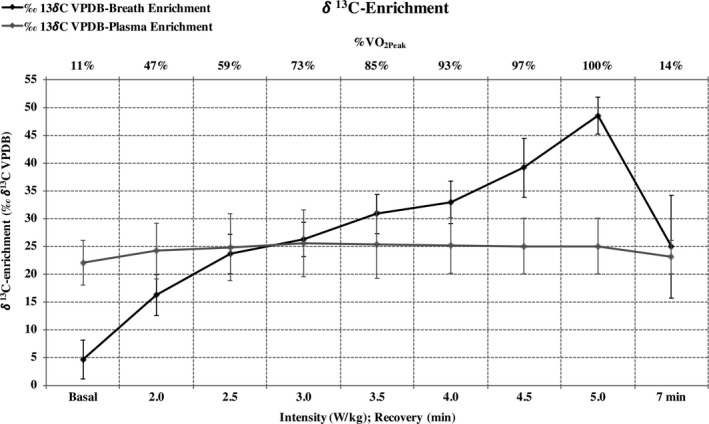
*δ* ^13^C‐breath and plasma enrichment thorough the aerobic test, for the whole range of aerobic intensities.

After the ^13^C labeled CHO repletion protocol, high *δ* ^13^C‐breath enrichment during the long‐graded laboratory test was obtained (4.7 ± 3.5 ‰ *δ* ^13^C VPDB, at the beginning of the exercise, to 48.5 ± 3.3 ‰ *δ* ^13^C VPDB when the subject reached exhaustion) (Fig. 2). Plasma ^13^C:^12^C ratios for free fatty acids, triglycerides, and proteins ranged from: −5.4 ± 4.9 to −5.2 ± 5.0 ‰ *δ* ^13^C VPDB, from −5.2 ± 3.8 to −6.1 ± 3.9 ‰ *δ* ^13^C VPDB, from −1.7 ± 1.9 to −1.5 ± 1.9 ‰ *δ* ^13^C VPDB; respectively, thorough the long‐graded test. In addition, plasma ^13^C:^12^C ratios for glucose are shown in Figure 2. UUNE measured during the protocol was 0.0096 ± 0.0003 g·min^−1^, corresponding to a protein oxidation rate of 0.0535 ± 0.0017 g·min^−1^.

### CHO_OxR_ and Fat_OxR_ differences between ^13^C:^12^C ratio technique respect to indirect calorimetry

**Figure 3:**
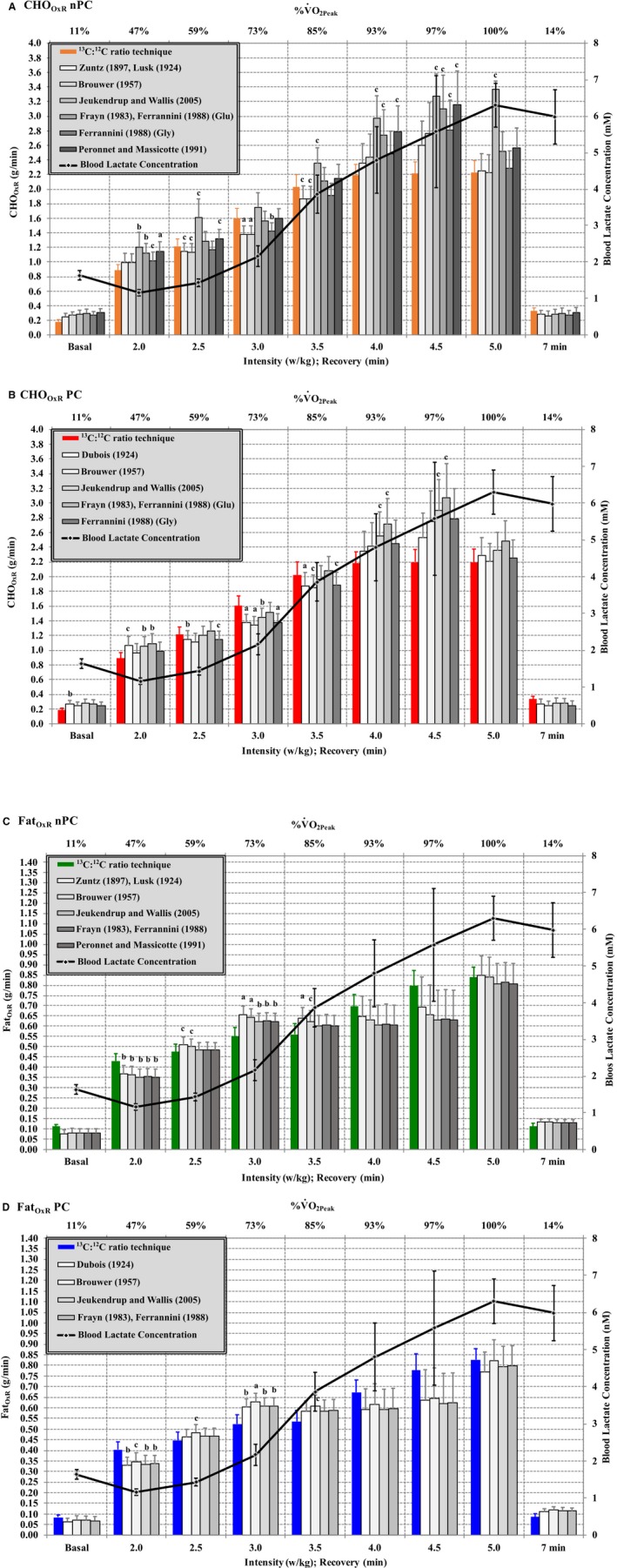
Fuel_OxR_ differences between indirect calorimetry respect to the method of reference ^13^C:^12^C ratio technique) at each specific intensity of exercise and during the recovery period, for CHO_O__xR_ nPC, CHO_O__xR_ PC, Fat_OxR_ nPC and Fat_OxR_ PC. PC: including the protein component, nPC: without the protein component (negligible).

The intensity during the long‐graded laboratory test ranged from 47 ± 3% to 99 ± 1% $\dot{V}$O_2Peak_ (2.0–5.0 W·kg^−1^). LT (1.5 ± 0.1 mmol/L; 2.6 ± 0.1 W·kg^−1^; 63.2 ± 2.9% $\dot{V}$O_2Peak_) and IAT (3.0 ± 0.1 mmol/L; 3.3 ± 0.1 W·kg^−1^; 75.0 ± 3.4% $\dot{V}$O_2Peak_) were determined (Fig. 3). These intensities ranged from 38 to 90%$\dot{V}$O_2max_ when considering the short‐graded and maximal laboratory test (Table 2). Significant differences were found between CHO_OxR_ between the method of reference when compared to several CHO_OxR_ equations, from the indirect calorimetry approach, and these differences were larger at intensities over IAT than under LT (Fig. 3A and B). FAT_OxR_ using the method of reference showed slight statistical significant differences in regards to many of the FAT_OxR_ equations, from indirect calorimetry, at intensities under LT also with some differences found over IAT (Fig. 3C and D). PC weight on CHO_OxR_ and Fat_OxR_ calculated with ^13^C:^12^C ratio technique induced small significant differences (*P *<* *0.001) between FAT_OxR_ PC (g·min^−1^) vs. FAT_OxR_nPC (g·min^−1^) (Basal: 0.08 ± 0.01 vs. 0.11 ± 0.01, 2.0 W·kg^−1^: 0.40 ± 0.04 vs. 0.43 ± 0.04, 2.5 W·kg^−1^: 0.45 ± 0.04 vs. 0.47 ± 0.04, 3.0 W·kg^−1^: 0.52 ± 0.04 vs. 0.55 ± 0.04, 3.5 W·kg^−1^: 0.54 ± 0.05 vs. 0.56 ± 0.05, 4.0 W·kg^−1^: 0.67 ± 0.06 vs. 0.70 ± 0.06, 4.5 W·kg^−1^: 0.78 ± 0.08 vs. 0.80 ± 0.07, 5.0 W·kg^−1^: 0.83 ± 0.05 vs. 0.84 ± 0.05, Rec 3 min: 0.13 ± 0.04 vs. 0.16 ± 0.04, Rec 5 min: 0.08 ± 0.01 vs. 0.11 ± 0.01, and Rec 7 min: 0.09 ± 0.01 vs. 0.11 ± 0.01, respectively), and no differences between CHO_OxR_ PC vs. nPC (Fig. 3).

**Table 2:** $\dot{V}$O_2_, $\dot{V}$CO_2_ and RER differences between 5th and 10th min of each stage of exercise (slow component effect)

Stage	Time	$\dot{V}$O_2peak_	$\dot{V}$O_2max_	$\dot{V}$O_2_				$\dot{V}$CO_2_				RER		
		Mean ± SE	Mean ± SE	Mean ± SE	Differences		*P*‐value	Mean ± SE	Differences		*P*‐value	Mean ± SE	Differences	*P*‐value
		(%)	(%)	(L·min^−1^)	(L·min^−1^)	(%)		(L·min^−1^)	(L·min^−1^)	(%)		(L·min^−1^)	(%)	
2.0 W·kg^−1^	5 min			1.45 ± 0.09				1.27 ± 0.08				0.87 ± 0.01		
	10 min	47 ± 3	38 ± 2	1.55 ± 0.10	0.10 ± 0.03	7.1 ± 1.8	NSD	1.34 ± 0.09	0.08 ± 0.02	6.2 ± 1.7	NSD	0.86 ± 0.01	−0.8 ± 0.3	NSD
2.5 W·kg^−1^	15 min			2.07 ± 0.13				1.75 ± 0.12				0.85 ± 0.01		
	20 min	59 ± 3	47 ± 3	1.95 ± 0.12	−0.12 ± 0.01	−5.6 ± 0.2	NSD	1.66 ± 0.11	−0.09 ± 0.01	−5.3 ± 0.2	NSD	0.85 ± 0.01	0.3 ± 0.1	NSD
3.0 W·kg^−1^	25 min			2.49 ± 0.12				2.11 ± 0.11				0.85 ± 0.01		
	30 min	73 ± 4	59 ± 3	2.45 ± 0.12	−0.04 ± 0.02	−1.7 ± 0.9	NSD	2.08 ± 0.11	−0.03 ± 0.02	−1.4 ± 0.7	NSD	0.85 ± 0.01	0.3 ± 0.2	NSD
3.5 W·kg^−1^	35 min			2.79 ± 0.10				2.43 ± 0.10				0.87 ± 0.01		
	40 min	85 ± 3	68 ± 2	2.81 ± 0.10	0.02 ± 0.02	0.8 ± 0.8	NSD	2.45 ± 0.10	0.02 ± 0.02	0.7 ± 0.8	NSD	0.87 ± 0.01	−0.1 ± 0.1	NSD
4.0 W·kg^−1^	45 min			3.14 ± 0.13				2.79 ± 0.14				0.89 ± 0.02		
	50 min	93 ± 2	78 ± 4	3.21 ± 0.15	0.07 ± 0.03	2.1 ± 0.9	NSD	2.87 ± 0.16	0.08 ± 0.03	2.4 ± 1.1	NSD	0.89 ± 0.02	0.3 ± 0.2	NSD
4.5 W·kg^−1^	55 min			3.30 ± 0.08				2.95 ± 0.13				0.90 ± 0.04		
	60 min	97 ± 2	88 ± 5	3.43 ± 0.09	0.13 ± 0.01	4.0 ± 0.3	NSD	3.11 ± 0.13	0.16 ± 0.02	5.3 ± 0.7	NSD	0.91 ± 0.04	1.2 ± 0.4	NSD
5.0 W·kg^−1^	65 min			3.32 ± 0.09				2.87 ± 0.12				0.86 ± 0.02		
	70 min	100 ± 1	90 ± 2	3.53 ± 0.10	0.21 ± 0.01	6.4 ± 0.4	NSD	3.05 ± 0.13	0.17 ± 0.01	6.0 ± 0.4	NSD	0.86 ± 0.02	−0.4 ± 0.4	NSD

### ^13^C:^12^C ratio technique and indirect calorimetry agreement for each CHO_OxR_ and Fat_OxR_ stoichiometric equation

Regarding the Deming regression used, estimates of the measurement error model for error‐variance ratio *λ = 1* with all the relationships being between the closest to the agreement line with intercept zero and slope one for each pair of equations (*a*_0_ + *b*_0_·*X*_*i*_ = 0 + 1·*X*_*i*_^0^): CHO_OxR_ nPC (*a*_0_ = 0.145 ± 0.014, *b*_0_ = 0.775 ± 0.039), CHO_OxR_ PC (*a*_0_ = 0.140 ± 0.017, *b*_0_ = 0.819 ± 0.027), Fat_OxR_ nPC (*a*_0_ = 0.064 ± 0.001, *b*_0_ = 0.883 ± 0.008), and Fat_OxR_ PC (*a*_0_ = 0.046 ± 0.001, *b*_0_ = 0.893 ± 0.008). Confidence intervals suggested an intercept different from zero and a slope different from one (*a*_0_≠0, *b*_0_≠1), thus the agreement intervals were made (i.e. fixed and proportional bias) (Liao and Capen 2011).

The level of agreement (Δ and %), for each pair of equations, is shown in Table 3. None of the pair of equations met a perfect agreement (95%, threshold *k* = 5%). For all CHO_OxR_ stoichiometric equations the agreement was moderate (51–75%) and poor for FAT_OxR_ equations (28–32%), when compared to the method of reference. The best agreement for CHO_OxR_ PC equations was the Jeukendrup and Wallis (2005) equation (75%) and Frayn (1983) and Ferrannini (1988) (Glu) equations (71%) for CHO_OxR_ nPC and for both, Fat_OxR_ PC and FAT_OxR_ nPC, all the equations showed similar agreement levels.

**Table 3:** Liao and Capen (2011) analyses: CHO_OxR_ and Fat_OxR_ pairs in agreement between ^13^C:^12^C ratio technique respect to indirect calorimetry to each stoichiometric equation. Fixed and proportional bias, and agreement intervals for each pair of equations assuming *λ* = 1

		Fixed and proportional bias	Δ	Agreement (%)	Agreement (*n*)
CHO nPC	SE_CHO_nPC__#1	0.095 + 0.897 x (*X*_*i*_)	[−0.200 0.200]	55	85
	SE_CHO_nPC__#3	0.146 + 0.833 x (*X*_*i*_)	[−0.286, 0.286]	66	101
	SE_CHO_nPC__#4	0.148 + 0.737 x (*X*_*i*_)	[−0.296, 0.296]	72	111
	SE_CHO_nPC__#5	0.133 + 0.827 x (*X*_*i*_)	[−0.271, 0.271]	65	100
	SE_CHO_nPC__#6	0.146 + 0.725 x (*X*_*i*_)	[−0.295, 0.295]	73	112
	SE_CHO_nPC__#7,#8	0.201 + 0.631 x (*X*_*i*_)	[−0.498, 0.498]	75	116
	Mean ± SE	0.145 + 0.775 x (*X*_*i*_)	[−0.308, 0.308] ± 0.041	68 ± 3	104 ± 5
CHO PC	SE_CHO_PC__#2	0.078 + 0.901 x (*X*_*i*_)	[−0.207, 0.207]	51	79
	SE_CHO_PC__#3	0.164 + 0.832 x (*X*_*i*_)	[−0.286, 0.286]	66	101
	SE_CHO_PC__#4	0.169 + 0.737 x (*X*_*i*_)	[−0.296, 0.296]	71	110
	SE_CHO_PC__#5	0.153 + 0.827 x (*X*_*i*_)	[−0.271, 0.271]	65	100
	SE_CHO_PC__#7,#8	0.134 + 0.798 x (*X*_*i*_)	[−0.282, 0.282]	68	105
	Mean ± SE	0.140 + 0.819 x (*X*_*i*_)	[−0.268, 0.268] ± 0.016	64 ± 4	99 ± 5
Fat nPC	SE_Fat_nPC__#1	0.060 + 0.845 x (*X*_*i*_)	[−0.031, 0.031]	28	43
	SE_Fat_nPC__#3	0.065 + 0.855 x (*X*_*i*_)	[−0.032, 0.032]	30	46
	SE_Fat_nPC__#4	0.063 + 0.884 x (*X*_*i*_)	[−0.031, 0.031]	28	43
	SE_Fat_nPC__#5	0.065 + 0.886 x (*X*_*i*_)	[−0.032, 0.032]	28	43
	SE_Fat_nPC__#6	0.065 + 0.886 x (*X*_*i*_)	[−0.032, 0.032]	28	43
	Mean ± SE	0.064 + 0.871 x (*X*_*i*_)	[−0.032, 0.032] ± 0.000	28 ± 0	44 ± 1
Fat PC	SE_Fat_PC__#2	0.048 + 0.903 x (*X*_*i*_)	[−0.031, 0.031]	29	45
	SE_Fat_PC__#3	0.045 + 0.870 x (*X*_*i*_)	[−0.032, 0.032]	32	49
	SE_Fat_PC__#4	0.045 + 0.900 x (*X*_*i*_)	[−0.031, 0.031]	29	45
	SE_Fat_PC__#6	0.046 + 0.900 x (*X*_*i*_)	[−0.032, 0.032]	30	46
	Mean ± SE	0.046 + 0.893 x (*X*_*i*_)	[−0.032, 0.032] ± 0.000	30 ± 1	46 ± 1

PC, including the protein component; nPC, without the protein component (negligible); SE_CHO_, Stoichiometric equation for CHO; SE_Fat_, Stoichiometric equation for Fat; Δ, agreement interval, number (*n*) and proportion (%) of pairs lying in agreement, estimated according to the measurement error model parameters estimated by setting *λ* = 1 and estimating it via random effect models.

The best fit polynomial regression model (cubic adjustment) between each pair of equations is shown in Table 4. The new error regression model improved, significantly, the mean slope for all CHO_OxR_ equations: from 0.775 ± 0.039 to 1.118 ± 0.021 for CHO_OxR_ nPC equations (*P *<* *0.01), and from 0.819 ± 0.027 to 1.099 ± 0.004 for CHO_OxR_ PC equations (*P *<* *0.001). However, the mean slopes for all FAT_OxR_ equations were not closer to 1 with this new model (from 0.883 ± 0.008 to 1.157 ± 0.002 for Fat_OxR_ nPC (*P *<* *0.001), and from 0.893 ± 0.008 to 1.160 ± 0.003 for Fat_OxR_ PC (*P *<* *0.001)). Thus, the systematic error of all CHO_OxR_ were improved using the model of this present study, however the agreement (random error) was not improved for this new model.

**Table 4:** Best fit polynomial regression model (cubic model adjustment) for each stoichiometric equation respect to ^13^C:^12^C ratio technique equations

		*R* ^2^	Best fit model	Cubic model adjustment vs. ^13^C:^12^C				Mean difference	
				Fixed and proportional bias	Δ	Agreement (%)	Agreement (n)	^13^C:^12^C vs. indirect calorimetry	^13^C:^12^C vs. adjustment
CHO nPC	SE_CHO_nPC__#1	0.858	*Y* = −0.033·X_i_^3^ + 0.069·X_i_^2^ + 0.971·X_i_ + 0.043	−0.096 + 1.090 x (X_i_)	[−0.134, 0.134]	40	62	0.03 ± 0.03	0.01 ± 0.03
	SE_CHO_nPC__#3	0.834	*Y* = −0.018·X_i_^3^ + 0.009 ·X_i_^2^ + 1.004·X_i_ + 0.047	−0.116 + 1.110 x (X_i_)	[−0.154, 0.154]	40	62	0.05 ± 0.04	0.01 ± 0.03
	SE_CHO_nPC__#4	0.842	*Y* = −0.014·X_i_^3^ + 0.020·X_i_^2^ + 0.876·X_i_ + 0.044	−0.101 + 1.090 x (X_i_)	[−0.150, 0.150]	42	64	0.19 ± 0.04	0.01 ± 0.03^a^
	SE_CHO_nPC__#5	0.844	*Y* = −0.020·X_i_^3^ + 0.028·X_i_^2^ + 0.962·X_i_ + 0.044	−0.108 + 1.101 x (X_i_)	[−0.147, 0.147]	42	65	0.07 ± 0.04	0.01 ± 0.03^c^
	SE_CHO_nPC__#6	0.844	*Y* = −0.014·X_i_^3^ + 0.023·X_i_^2^ + 0.854·X_i_ + 0.043	−0.103 + 1.097 x (X_i_)	[−0.147, 0.147]	43	66	0.22 ± 0.56	0.01 ± 0.36^a^
	SE_CHO_nPC__#7,#8	0.719	*Y* = 0.032·X_i_^3^ − 0.339·X_i_^2^ + 1.410·X_i_ − 0.066	−0.236 + 1.222 x (X_i_)	[−0.249, 0.249]	47	72	0.32 ± 0.06	0.01 ± 0.04^a^
	Mean ± SE	0.824	*Y* = −0.011·X_i_^3^ − 0.032·X_i_^2^ + 1.013·X_i_ + 0.026	−0.127 + 1.118 x (X_i_)	[−0.164, 0.164] ± 0.017	42 ± 1	65 ± 2	0.15 ± 0.05	0.01 ± 0.00
CHO PC	SE_CHO_PC__#2	0.845	*Y* = −0.047·X_i_^3^ + 0.166·X_i_^2^ + 0.799·X_i_ + 0.088	−0.104 + 1.098 x (X_i_)	[−0.145, 0.145]	40	62	0.03 ± 0.03	0.00 ± 0.03
	SE_CHO_PC__#3	0.833	*Y* = −0.018·X_i_^3^ + 0.011·X_i_^2^ + 0.998·X_i_ + 0.070	−0.112 + 1.104 x (X_i_)	[−0.156, 0.156]	41	63	0.02 ± 0.04	0.00 ± 0.03
	SE_CHO_PC__#4	0.841	*Y* = −0.014·X_i_^3^ + 0.021·X_i_^2^ + 0.872·X_i_ + 0.070	−0.098 + 1.086 x (X_i_)	[−0.151, 0.151]	42	65	0.16 ± 0.04	0.01 ± 0.03^a^
	SE_CHO_PC__#5	0.843	*Y* = −0.020·X_i_^3^ + 0.029·X_i_^2^ + 0.958·X_i_ + 0.069	−0.104 + 1.097 x (X_i_)	[−0.148, 0.148]	42	65	0.04 ± 0.04	0.00 ± 0.03
	SE_CHO_PC__#7,#8	0.838	*Y* = −0.020·X_i_^3^ + 0.040·X_i_^2^ + 0.902·X_i_ + 0.053	−0.116 + 1.111 x (X_i_)	[−0.150, 0.150]	41	63	0.10 ± 0.04	0.00 ± 0.03^a^
	Mean ± SE	0.840	*Y* = −0.024·X_i_^3^ + 0.053·X_i_^2^ + 0.906·X_i_ + 0.070	−0.107 + 1.099 x (X_i_)	[−0.150, 0.150] ± 0.002	41 ± 0	64 ± 1	0.07 ± 0.03	0.00 ± 0.00
Fat nPC	SE_Fat_nPC__#1	0.783	*Y* = −0.577·X_i_^3^ + 1.122·X_i_^2^ + 0.240·X_i_ + 0.128	−0.056 + 1.150 x (X_i_)	[−0.122, 0.122]	19	29	0.00 ± 0.01	0.00 ± 0.01
	SE_Fat_nPC__#3	0.774	*Y* = −0.588·X_i_^3^ + 1.111·X_i_^2^ + 0.271·X_i_ + 0.129	−0.059 + 1.157 x (X_i_)	[−0.022, 0.022]	20	31	−0.01 ± 0.01	0.00 ± 0.01
	SE_Fat_nPC__#4	0.774	*Y* = −0.640·X_i_^3^ + 1.176·X_i_^2^ + 0.279·X_i_ + 0.129	−0.059 + 1.156 x (X_i_)	[−0.022, 0.022]	20	31	−0.02 ± 0.01	0.00 ± 0.01^a^
	SE_Fat_nPC__#5	0.770	*Y* = −0.627·X_i_^3^ + 1.148·X_i_^2^ + 0.294·X_i_ + 0.130	−0.061 + 1.161 x (X_i_)	[−0.023, 0.023]	20	31	−0.02 ± 0.01	0.00 ± 0.01^a^
	SE_Fat_nPC__#6	0.770	*Y* = −0.627·X_i_^3^ + 1.148·X_i_^2^ + 0.294·X_i_ + 0.130	−0.061 + 1.161 x (X_i_)	[−0.023, 0.023]	20	31	−0.02 ± 0.01	0.00 ± 0.01^a^
	Mean ± SE	0.774	*Y* = −0.612·X_i_^3^ + 1.141·X_i_^2^ + 0.276·X_i_ + 0.129	−0.059 + 1.157 x (X_i_)	[−0.042, 0.042] ± 0.020	20 ± 0	31 ± 0	−0.01 ± 0.00	0.00 ± 0.00
Fat PC	SE_Fat_PC__#2	0.775	*Y* = −0.528·X_i_^3^ + 0.905·X_i_^2^ + 0.450·X_i_ + 0.098	−0.055 + 1.157 x (X_i_)	[−0.023, 0.023]	18	27	−0.02 ± 0.01	0.00 ± 0.01^b^
	SE_Fat_PC__#3	0.776	*Y* = −0.567·X_i_^3^ + 1.032·X_i_^2^ + 0.345·X_i_ + 0.101	−0.055 + 1.170 x (X_i_)	[−0.022, 0.022]	18	28	0.00 ± 0.01	0.00 ± 0.01
	SE_Fat_PC__#4	0.777	*Y* = −0.612·X_i_^3^ + 1.076·X_i_^2^ + 0.366·X_i_ + 0.101	−0.054 + 1.154 x (X_i_)	[−0.023, 0.023]	18	28	−0.01 ± 0.01	0.00 ± 0.01^c^
	SE_Fat_PC__#6	0.772	*Y* = −0.608·X_i_^3^ + 1.072·X_i_^2^ + 0.365·X_i_ + 0.102	−0.055 + 1.159 x (X_i_)	[−0.023, 0.023]	19	29	−0.01 ± 0.01	0.00 ± 0.01^c^
	Mean ± SE	0.775	*Y* = −0.579·X_i_^3^ + 1.021·X_i_^2^ + 0.382·X_i_ + 0.101	−0.055 + 1.160 x (X_i_)	[−0.023, 0.023] ± 0.000	18 ± 0	28 ± 0	−0.01 ± 0.00	0.00 ± 0.00

PC, with the protein component; nPC, without the protein component (negligible), number (*n*) and proportion (%) of pairs lying in agreement, estimated according to the measurement error model parameters estimated by setting *λ *= 1 and estimating it via random effect models.

^a^ *P* < 0.001, ^b^ *P* < 0.01, ^c^ *P* < 0.05.

## Discussion

Most of the subjects of this study performed an extenuating physical effort to achieve the goal of the depletion protocol (with Borg's scale readings of 19.9 ± 0.2). In addition, none of the subjects reported any gastrointestinal disturbances in relation to the carbohydrate repletion protocol. The nutritional intervention of this study was successful in enriching the endogenous carbohydrate stores (Fig. 2) and *δ* ^13^C‐breath enrichment was similar to previously carried out studies using similar methodologies (i.e. 48.5 ± 3.7 vs. 51.0 ± 1.5 *δ*‰ VPDB) (Romijn et al. 1992). The step‐length used in the long‐graded laboratory test was considered long enough to reach a metabolic steady state (Robergs 2018) where the intensities ranged from 38 to 90%$\dot{V}$O_2max_. In regards to the long‐graded and maximal laboratory test, the slow component increased by less than 10% at each intensity of exercise (Table 2). In this case, the ^13^C was considered representative of the metabolic response for each intensity (Romijn et al. 1992). *δ* ^13^C‐breath enrichment increased concomitantly with the intensity of exercise, as Trimmer et al. (2001) have suggested previously. Most of the ^13^CO_2_ breath tests involve the oral administration of a carbon labeled substrate releasing ^13^C in its metabolic pathway (Lefebvre 1985). However, a number of factors interfere with different steps in the ^13^C metabolic route which can subsequently affect the rate of appearance in exhaled ^13^CO_2_. These factors are: (a) the isotopic dilution: after oxidation of ^13^C‐glucose, the ^13^CO_2_ produced mixes with the bicarbonate pool, which has a slow turnover rate producing an isotopic dilution and delay in the breath ^13^CO_2_ rate of appearance; (b) the background ^13^C enrichment: the current ^13^C exogenous substrate ingested modifies the ^13^CO_2_ composition provided from endogenous substrate stores, inducing large overestimation of ^13^C recovery in the expired ^13^CO_2_ (Pallikarakis et al. 1991). These limitations were controlled in this study, using the ^13^C:^12^C ratio technique, which avoids isotopic dilution estimating substrates oxidation independently from the *inaccurate* $\dot{V}$CO_2_ (Eqs. (6) and (7)) (see Romijn et al. (1992) for more information regarding the bases and limitations of ^13^C:^12^C ratio technique). The ^13^C background enrichment was homogenized by means of the glycogen stores depletion‐repletion procedure whilst assuming that primary glycogen stores, skeletal‐muscles and liver, were successfully labeled with the same amount of ^13^C (Romijn et al. 1992). In this regards, authors have previously used a similar depletion protocol, to the one reported in this presented study, which also resulted in near‐complete depletion of glycogen stores in *vastus lateralis* muscle as measured by biopsy (Coyle et al. 1986). However, one of the limitations of the present study was the inability to verify the level of glycogen stores depletion, in skeletal muscles, by means biopsy. Nonetheless, most of the variability of the results in this study attributed to the metabolic changes induced by the intensity/duration of exercise. Several, authors have previously used ^13^CO_2_ breath tests to estimate fuel oxidation rates during the exercise with the majority, however, using inaccurate calculations for calculating $\dot{V}$CO_2_ values (Decombaz et al. 1985; Massicotte et al. 1986, 1992; Guezennec et al. 1989; Peronnet et al. 1990; Saris et al. 1993; Sonko et al. 1993; Wagenmakers et al. 1993; Jeukendrup et al. 1996; Rocker et al. 1996; Riddell et al. 2000; Trimmer et al. 2001; van Loon et al. 2005; Rowlands et al. 2008; Roberts et al. 2014); in spite of the effort of these authors trying to control the ^13^C isotopic dilution equilibrating by means of prolonged steady‐state periods (>1 h) (Pallikarakis et al. 1991) and the ^13^C background enrichment (Wagenmakers et al. 1993). This is the first study, that we are aware of, studying the agreement between indirect calorimetry, in comparison to a method of reference, for a wide range of aerobic intensities and providing detailed analysis on both the relative and absolute reliability of each stoichiometric equation. For the above mentioned reasons the results of this study were difficult to compare, in depth, with others.

Some authors have suggested that fuels_OxR_ variability between different stoichiometric equations is small (Frayn 1983; Ferrannini 1988; Peronnet et al. 1990; Jeukendrup and Wallis 2005), between ~5% and 6% CHO_OxR_ and ~3–6% between FAT_OxR_ equations for a moderate metabolic response ($\dot{V}$O_2_ = 2.5 L·min^−1^ and RER = 0.9) (Romijn et al. 1992; Jeukendrup and Wallis 2005). Some authors (Romijn et al. 1992) have reported good relative reliability, using systematic error by means of a paired *t*‐test (Atkinson and Nevill 1998) approach, for both CHO_OxR_ and Fat_OxR_ at a specific intensities of exercise (80–85%$\dot{V}$O_2max_), between indirect calorimetry and the ^13^C:^12^C ratio technique. In this study, differences were higher for most of the intensities, especially over the IAT, when fuel_OxR_ was compared between indirect calorimetry and the method of reference for all equations (Fig. 3). Although agreement intervals (Δ) were very demanding and narrow, for each group of equations, one important finding of this study was that none of the stoichiometric equations met a perfect concordance (i.e. proportion of agreement (%)) (Liao and Capen 2011) between indirect calorimetry when compared to the method of reference (Table 3). When measuring, there are two types of error which explain the variability of the measurements: the aleatory (random error) and the systematic error (bias)) (Atkinson and Nevill 1998). The main sources of systematic error, in this study, were attributed to the different coefficients of fuel used for each stoichiometric equation (Frayn 1983; Ferrannini 1988; Peronnet et al. 1990) and in relation to inherent instrument error (indirect calorimetry, and mass spectrometry (~0.1%, almost negligible)). The Oxycon Pro^®^ has been previously validated against the Douglas bags method (Carter and Jeukendrup 2002; Macfarlane and Wong 2012; Foss and Hallen 2018), demonstrating that Oxycon Pro^®^ produces $\dot{V}$E, $\dot{V}$O_2_ and $\dot{V}$CO_2_ values that are very similar to Douglas bags method (systematic error close to 0, and small aleatory error $\dot{V}$E (±5 L·min^−1^), $\dot{V}$O_2_ (±0.1%) and $\dot{V}$CO_2_ (±0.2%)) (Foss and Hallen 2018). Taking this into account the author, of this present study, deem the indirect calorimeter used as valid and reliable for the wide range of aerobic intensities used. However, the author to note that one of the limitations of this study was not to use the Douglas bags method (often considered as the gold standard) to compare the gas exchange values in comparison to the ^13^C:^12^C ratio technique.

The main aleatory error, in this study, was attributed to the individual response to non‐metabolic CO_2_ production and metabolic CO_2_ losses during the exercise (i.e. cutaneous gas exchange, high‐energy phosphates use, glyconeogenesis, desaturation of fatty acids, size of urea pool, colon microbial metabolism and Cori cycle (Frayn 1983), and leucine oxidation (Wolfe and Jahoor 1990). In this present study, while the systematic and aleatory errors remained small through all the intensities of exercise for all the FAT_OxR_ equations (Table 3), for CHO_OxR_ equations both errors were larger (Table 3). In view of these findings, it seems that the non‐metabolic CO_2_ measured by indirect calorimetry overestimates, considerably, CHO_OxR_ and underestimates FAT_OxR_ with compared to the method of reference used, especially at exercise intensities over IAT (Fig. 3). This has also been previously reported (Romijn et al. 1992). At moderate to high aerobic intensities $\dot{V}$CO_2_ increases concomitantly with intensity of exercise due to the increment of metabolic CO_2_ coming from endogenous fuel_OxR_ and to the increment of non‐metabolic CO_2_ released from the bicarbonate pool with effect of metabolic acidosis buffering (Barstow et al. 2018) and this increment is higher when exercising over IAT (Gonzalez‐Haro 2011). For these reasons, in particular regards to CHO_OxR_, at moderate to high intensities the error of measurement is higher (Table 3).

This study has been successful in determining the fuels_OxR_ differences between a widely and routinely used indirect calorimeter (validated against the Douglas bags method) in comparison to the ^13^C:^12^C ratio technique. The theoretical error, expressed in g·min^−1^, introduced when the $\dot{V}$CO_2_ is measured by means of indirect calorimetry is shown in Figure 3. While the error is small for Fat_OxR_ and for CHO_OxR_ at low intensities, the error is not insignificant for CHO_OxR_ at moderate and high aerobic intensities. In addition, the most important finding yielded in this study was the Jeukendrup and Wallis (2005) equation for CHO_OxR_ nPC, Frayn (1983) and Ferrannini (1988) (Glu) equations for CHO_OxR_ PC which showed the best agreement levels (Table 3) to findings reported in this present study. These equations used different assumptions, while Frayn (1983) and Ferrannini (1988) (Glu) are based on glucose coefficients, Jeukendrup and Wallis (2005) equation is based on a mixture of glucose and glycogen coefficients. The fact that it had closer agreements is likely due to using glucose oxidation exclusively which, in‐turn, is unlikely to introduce a major CHO_OxR_ error during exercise (Ferrannini 1988) (i.e. stoichiometry for glucose ~10% higher than for glycogen (Jeukendrup and Wallis 2005)). However, this study demonstrates that the equations, using a mixture of glucose and glycogen coefficients, show better agreements, especially when the protein component is considered negligible. However, the mixture coefficient used by Jeukendrup and Wallis (Jeukendrup and Wallis 2005) is arbitrary because the contribution of muscle glycogen to carbohydrate oxidation varies from 0% to 78% (Harvey et al. 2007) and other mixtures might be proposed in the future to try to improve CHO_OxR_ during the exercise. All the Fat_OxR_ equations showed similar concordances and can be used indistinctly (Table 3). Peronnet et al. (1990) and Jeukendrup and Wallis (2005) equations were based on a coefficient calculated from the average of 13 fatty acids (FA) (C_17.2702_H_32.7142_O_2_) representing 99% of FA at the adipose tissue. In this study, UUNE was measured to estimate the PC (Ferrannini 1988). Most of the time, stoichiometric equations are applied to estimate FAT_OxR_ and CHO_OxR_ routinely using indirect calorimetry, whilst considering PC to be negligible, in order to facilitate its calculation (Harvey et al. 2007). PC had no effect on CHO_OxR_, however it provoked significantly lower FAT_OxR_ (4 ± 2%) during all exercise intensities (Fig. 3). These results are in agreement with previous studies who have reported amino acid oxidation potentially contributing up to 10%, or more, of total substrate utilization in prolonged exercise (Wagenmakers 1998).

Another important contribution of this research is that it has provided a mathematical adjustment to successfully reduce the systematic error of fuel_OxR_ from indirect calorimetry, especially when CHO_OxR_ is estimated above the IAT. This novel mathematical approach is very useful to all sport/exercise physiologists around the world who use indirect calorimetry as a routine tool to assess the metabolic response to training and nutritional interventions. It is important to consider, while the systematic error can be reduced through the use of such mathematical approaches, the aleatory error is very difficult to remove or control (Atkinson and Nevill 1998). Thus, this mathematical adjustment is useful to minimize the systematic error but is not able to reduce the aleatory error inherent with the indirect calorimetry approach.

### Perspective

This is the first study in the available scientific literature which has evaluated the agreement levels of commonly used stoichiometric equations, as used in indirect calorimeters, in comparison to an advanced method of reference (the ^13^C:^12^C ratio technique), for a wide range of aerobic intensities during an incremental test with 10 min stage duration. None of the stoichiometric equations showed a perfect agreement. The Jeukendrup and Wallis (2005) equation showed the best concordance (75%) for CHO_OxR_ nPC equations whilst the Frayn (1983) and Ferrannini (1988) (Glu) equations showed the best concordance (71%) for CHO_OxR_ PC equations. This study provided an adjustment to reduce the systematic error between the true value (via the method of reference used in this present study) via the comparison of values to estimated values obtained through stoichiometric equations (i.e. correction for Jeukendrup and Wallis (2005): *Y* = 0.032·*X*_*i*_^3^−0.339·*X*_*i*_^2^ + 1.410·*X*_*i*_−0.066; and for Frayn (1983) and Ferrannini (1988) (Glu): *Y* = −0.014·*X*_*i*_^3^ + 0.021·*X*_*i*_^2^ + 0.872·*X*_*i*_ + 0.070, being Y the adjusted value and *X*_*i*_ the observed value thorough the stoichiometric equations). In light of the results, we are of the opinion that this study helps improve fuel_OxR_ accuracy when measured during exercise, especially in relation to CHO_OxR_, and helps reduce the associated systematic error of estimations inherent with the indirect calorimetry approach. This contribution will help in several fields of knowledge: sport/exercise physiology, sports/exercise nutrition, animal physiology etc. The most important difficulty found in this study lies in relation to indirect calorimeters which are not able to distinguish between metabolic and non‐metabolic CO_2_. Non‐metabolic CO_2_ production is the most important contributor to aleatory error and, unfortunately, that error cannot be corrected by any mathematical adjustments. Stoichiometric equations, together with indirect calorimetry, is a methodology used for more than one century and by taking into account both, the results of the present study and the current technological development, we are of the opinion that new methodologies should be developed to better measure metabolic CO_2_ in order to then estimate CHO_OxR_ and Fat_OxR_ during aerobic exercise. Finally, the results of this study are applicable for 10 min exercise durations and it would be useful to replicate this study assessing the non‐metabolic $\dot{V}$CO_2_ influence on fuel_OxR_ during longer exercise duration.

## Conflict of Interest

None declared.
